# Impact of Preoperative Statin Augmentation on Myocardial Injury Assessed by Biomarkers and Strain Parameters in Off-Pump Coronary Artery Bypass Grafting

**DOI:** 10.7759/cureus.101353

**Published:** 2026-01-12

**Authors:** Sudesh Prajapathi, Aditya Kapoor, Aditi Mohta, Surendra K Agarwal, Prabhat Tewari, Shantanu Pande, Bipin Chandra, Ankit Sahu, Roopali Khanna, Sudeep Kumar, Naveen Garg, Satyendra Tewari, Archana Sinha

**Affiliations:** 1 Department of Cardiology, All India Institute of Medical Sciences, Bhopal, IND; 2 Department of Cardiology, Sanjay Gandhi Postgraduate Institute of Medical Sciences, Lucknow, IND; 3 Department of Community Medicine, People’s College of Medical Sciences and Research Centre, People’s University, Bhopal, IND; 4 Department of Cardiovascular and Thoracic Surgery, Sanjay Gandhi Postgraduate Institute of Medical Sciences, Lucknow, IND; 5 Department of Cardiac Anesthesia, Sanjay Gandhi Postgraduate Institute of Medical Sciences, Lucknow, IND; 6 Department of Dietetics, Sanjay Gandhi Postgraduate Institute of Medical Sciences, Lucknow, IND

**Keywords:** atrial fibrillation, global longitudinal strain, hydroxymethylglutaryl-coa reductase inhibitors, myocardial ischemia, off-pump coronary artery bypass, rosuvastatin calcium

## Abstract

Background:Myocardial injury is common after off-pump coronary artery bypass grafting (OPCABG). Statins exert cardioprotection against ischemia/reperfusion injury through diverse pathways. We aimed to assess whether preoperative high-dose statin therapy enhances cardioprotection against ischemia/reperfusion injury in chronic statin users undergoing OPCABG.

Methods: In this parallel-group non-randomized observational study, patients receiving chronic statin therapy for more than 30 days were included, regardless of the type or dosage of statin used. Patients were assigned either to a rosuvastatin-loaded group (single dose of 40 mg, administered orally seven days before surgery) or to a comparator control group without any loading. Cardiac biomarkers, including troponin I (TnI), creatine kinase-MB (CK-MB), and N-terminal pro B-type natriuretic peptide (NT-proBNP), were assessed preoperatively and at 8, 24, and 48 hours after surgery. Global left ventricular strains (circumferential (GCS), longitudinal (GLS), and radial (GRS)) were evaluated preoperatively and at 48 hours and 30 days postoperatively.

Results: In this exploratory analysis, the loaded group showed significantly lower postoperative cardiac biomarker levels at all time points. All global left ventricular strains (GLS, GCS, and GRS) declined 48 hours post-surgery, but were significantly better in the loaded group. Global strains recovered until 30 days post-surgery, with higher recovery seen in the loaded group. Postoperative atrial fibrillation (AF) occurred less frequently in the loaded group (8% versus 24%, p = 0.001). On multivariable analysis, 48-hour GCS independently predicted poor postoperative left ventricular ejection fraction (LVEF) (≤50%) at 30 days. Receiver operating characteristic (ROC) analysis identified 48-hour GRS < 26.5% as the best predictor of poor postoperative LVEF (≤50%) at 30 days, with 89.5% sensitivity and 71.0% specificity.

Conclusion: Preoperative high-dose rosuvastatin confers significant cardioprotection in chronic statin users undergoing OPCABG, as reflected by reduced biomarker release, enhanced myocardial strain recovery, and lower incidence of atrial fibrillation. The observed association between statin loading and improved myocardial performance warrants confirmation in larger prospective studies.

## Introduction

The systemic inflammatory response due to ischemia/reperfusion in patients undergoing cardiopulmonary bypass during coronary artery bypass grafting (CABG) is a worrisome phenomenon well-known to surgeons and anesthesiologists [1]. This inflammatory cascade increases the risks of postoperative ischemic events, which can be fatal [2]. Annually, approximately 1.7 million cardiac procedures happen worldwide and are associated with a 30-day postoperative mortality rate of 3%-4% [3]. Given the magnitude of this burden and the central role of inflammation and thrombosis in postoperative complications, strategies that can modulate these pathways are of considerable clinical interest.

One such strategy involves statin therapy. Statins are a class of lipid-lowering medications that have gained attention for their potential benefits in cardiovascular procedures. While their primary function is cholesterol reduction, statins also show pleiotropic properties that protect against thrombosis, inflammation, and oxidative stress. Statins can thus emerge as promising agents to mitigate the perioperative risks associated with CABG [4-6].

Rationale behind statin loading

The pleiotropic effects of statins that confer protection against ischemia/reperfusion injury are mediated through the activation of antiapoptotic pathways (PI3K/Akt) [7]. In chronic statin users, these pleiotropic effects are attenuated due to the activation of certain cellular mechanisms that inactivate Akt. Animal studies have suggested that statin loading prior to ischemia/reperfusion injury can restore this cardioprotection through pleiotropic effects [8]. Studies have suggested beneficial effects of statin loading on infarct size in patients undergoing coronary angioplasty in comparison to chronic statin therapy [9,10]. Patients with CABG are at high risk of adverse cardiovascular events post-surgery due to ischemia/reperfusion injury. An extra dose of statin in these patients may recapture cardioprotection, reduce perioperative myocardial injury, and improve outcomes.

Recent studies have highlighted the favorable impact of preoperative statin therapy, particularly rosuvastatin, on the cardiac biomarker kinetics during off-pump CABG (OPCABG) [11]. Literature supports its role in reducing myocardial injury and modulating biomarker release patterns. Despite these encouraging findings, the practice of discontinuing statin therapy prior to CABG remains common, primarily due to concerns related to potential adverse effects exacerbated by surgical stress [2].

In light of the potential benefits of statins beyond lipid lowering and the existing gaps in knowledge, the present observational study aimed to investigate whether high-dose statin therapy administered shortly before OPCABG effectively “recaptures” cardioprotection in patients already on chronic statin therapy. The study’s primary objective was to identify the association of high-dose statin pretreatment with global left ventricular strain patterns in such patients undergoing OPCABG. The secondary objectives were to identify the association of preoperative high-dose statin therapy with cardiac biomarker levels post-CABG in both groups, to identify the predictive value of strain parameters on postoperative left ventricular dysfunction (left ventricular ejection fraction (LVEF) ≤ 50%) at 30 days, and to compare other clinically important parameters between the groups, such as hospital stay, intensive care unit (ICU) stay, ventilator duration, and inotrope duration, and cardiovascular outcomes, including mortality.

## Materials and methods

Study design and patient population

This parallel-group prospective observational study was conducted between December 2020 and December 2021 at a tertiary care center in India. The study included patients who had been on chronic statin therapy for more than 30 days, regardless of the type or dosage of statin used. Exclusion criteria were as follows: renal failure (defined as serum creatinine levels exceeding 3 mg/dL), active liver disease or elevated liver enzyme levels, left ventricular ejection fraction (LVEF) below 35%, history of muscle disease, known intolerance to statins, concomitant valve procedure, and presence of other comorbid illnesses. Assuming an alpha error of 0.05, power of 90%, and effect size (d) of 0.96, the sample size was obtained as 24 per group using G Power software version 3.1 [11]. Patients were consecutively enrolled in a 1:1 ratio into the statin-loaded group and the comparator non-loaded group, which served as the control, until the required sample size was achieved. Patients in the “loaded group” had received a single oral loading dose of rosuvastatin 40 mg seven days prior to CABG. The “control group” had continued with their previously prescribed statin regimen. The investigators conducting postoperative assessments were unaware of the study groups. The study was conducted in accordance with the institutional ethical standards and the Helsinki Declaration. Written informed consent was obtained from all patients. This study was approved by the Institutional Ethics Committee of Sanjay Gandhi Postgraduate Institute of Medical Sciences, Lucknow.

Study flow and data collection

All study participants underwent a comprehensive preoperative evaluation, including a detailed medical history, physical examination, complete hemogram, lipid profile, kidney function tests, electrocardiogram (ECG), and echocardiography (ECHO). Prior to CABG, biomarker levels including creatine kinase-MB (CK-MB), troponin I (TnI), and N-terminal pro-B-type natriuretic peptide (NT-proBNP) were measured for all patients, using fluorescence immunoassay with a commercially available kit (Alere Triage Cardio 3 panel; Alere San Diego, Inc., San Diego, CA). These biomarker measurements were repeated at 8, 24, and 48 hours after CABG. Additionally, 48 hours and 30 days post-surgery, all patients underwent two-dimensional echocardiography (2D ECHO) to assess global left ventricular strain, comprising global longitudinal strain (GLS), global circumferential strain (GCS), and global radial strain (GRS).

Speckle Tracking Strain Analysis

Global longitudinal strain (GLS) was calculated by averaging segmental strain values derived from three standard apical views: apical four-chamber (A4CH), apical two-chamber (A2CH), and apical parasternal long-axis (APLAX). Global circumferential strain (GCS) and global radial strain (GRS) were derived from two parasternal short-axis (PSAX) views obtained at the mitral valve and papillary muscle levels. For GCS and GRS, regional strain values from 12 myocardial segments (six segments from each PSAX level) were averaged. All echocardiographic acquisitions and strain analyses were performed using a Vivid 7 echocardiography system (GE Healthcare, Waukesha, WI) by a single trained cardiologist, blinded to group allocation, to eliminate inter-observer variability.

All patients were followed up one month post-surgery for a comprehensive examination, and mortality and cardiovascular outcomes in both groups were recorded. Additionally, other relevant parameters, such as hospital stay, ICU stay, ventilator duration, and inotrope duration, were also recorded. All patients received standard perioperative atrial fibrillation (AF) prophylaxis with beta-blockers as per institutional protocol unless contraindicated, while amiodarone was not used prophylactically and was reserved for treatment of postoperative atrial fibrillation, with similar use across both groups.

Statistical analysis

Data was analyzed using IBM SPSS version 22 (IBM Corp., Armonk, NY). Categorical variables were presented as frequencies and percentages, and continuous variables were presented in terms of mean and standard deviation (SD). After normality testing, Student’s t-test was used to compare the means between groups. The chi-square test was used to compare proportions between groups. To identify the association and predictive value of strain parameters at 48 hours toward postoperative LV dysfunction (LVEF ≤ 50%) at 30 days, univariable and multivariable logistic regression analyses were done, and receiver operating characteristic (ROC) curves were plotted. A p-value of less than 0.05 was considered statistically significant. The Youden index (= sensitivity + specificity - 1) was calculated to determine the optimal cutoff values of strain parameters.

## Results

**Figure 1:**
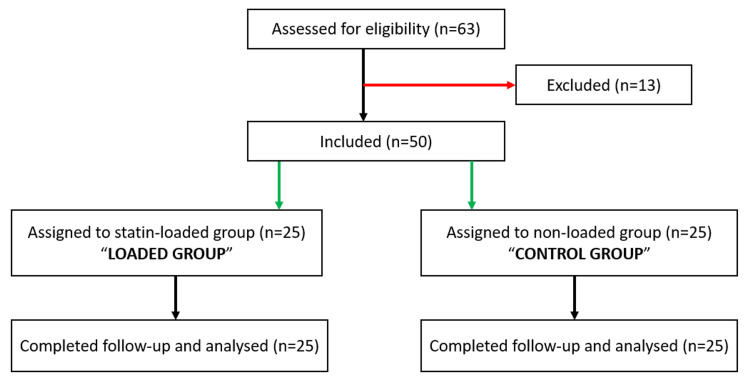
Study flow

A total of 63 patients were screened. Two patients were excluded due to renal dysfunction, two due to liver dysfunction, four due to poor LVEF, and five due to concomitant valve procedure. Finally, 25 patients were included in both the loaded and the control group (Figure 1).

The baseline characteristics (Table 1) indicate that the groups were well-matched, with no statistically significant differences in any variable (all p-values > 0.05). The mean age was 61.84 ± 8.45 years in the loaded group and 58.68 ± 7.32 years in the control group (p = 0.16). Both groups had a similar sex distribution, with mostly male patients (n = 21, 84% in the loaded group; n = 22, 88% in the control group). The prevalence of comorbidities and risk factors such as acute coronary syndrome (ACS), chronic stable angina (CSA), diabetes mellitus, hypertension, and smoking was similar in both groups. Laboratory parameters, including hemoglobin, serum creatinine, and lipid profile components, were also comparable between the two groups.

**Table 1:** Baseline characteristics of patients who underwent OPCABG Data are represented as number (%) and mean ± SD. p < 0.05 was considered statistically significant. OPCABG: off-pump coronary artery bypass grafting, ACS: acute coronary syndrome, BMI: body mass index, CSA: chronic stable angina, HDL: high-density lipoprotein, LDL: low-density lipoprotein, VLDL: very low-density lipoprotein, BP: blood pressure, SD: standard deviation

Baseline variables	Loaded group (n = 25)	Control group (n = 25)	p-value
Age (years)	61.84 ± 8.45	58.68 ± 7.32	0.16
Male	21 (84)	22 (88)	0.68
Female	4 (16)	3 (12)	
BMI (kg/m^2^)	26.72 ± 1.70	26.64 ± 2.10	0.88
ACS	9 (36)	9 (36)	1.00
CSA	16 (64)	16 (64)	
Diabetes mellitus	15 (60)	14 (56)	0.77
Hypertension	20 (80)	19 (76)	0.73
Smoking	13 (52)	14 (56)	0.78
Systolic BP (mmHg)	128.32 ± 16.54	124 ± 18.71	0.39
Diastolic BP (mmHg)	73.08 ± 9.93	70.80 ± 9.09	0.40
Hemoglobin (g/dL)	11.60 ± 0.92	11.80 ± 1.08	0.49
Serum creatinine (mg/dL)	1.13 ± 0.22	1.06 ± 0.16	0.24
Total cholesterol (mg/dL)	143.80 ± 18.02	142.24 ± 18.31	0.76
HDL (mg/dL)	29.40 ± 4.32	28. 92 ± 4.21	0.69
LDL (mg/dL)	84.16 ± 20.11	84.88 ± 22.02	0.92
VLDL (mg/dL)	26.04 ± 3.08	26.48 ± 2.57	0.58
Triglycerides (mg/dL)	135.80 ± 8.79	134.32 ± 16.63	0.70

Table 2 summarizes the biochemical, strain, and other parameters of patients who underwent OPCABG. The hospital stay duration, intensive care unit (ICU) stay duration, ventilator duration, and inotrope duration were comparable between the groups. Echocardiographic parameters showed better preservation of myocardial function in the loaded group. The GLS was significantly better in the loaded group at 48 hours (-14.08 ± 1.19 % versus -12.00 ± 1.38 %, p < 0.01) and 30 days (-16.80 ± 1.66 % versus -14.28 ± 1.21 %, p < 0.01). GCS was better in the loaded group at 48 hours (-16.48 ± 2.58 % versus -14.76 ± 2.38 %, p = 0.02), while GRS showed improvement at 48 hours (27.20 ± 6.12 % versus 21.92 ± 6.05 %, p < 0.01). These values reflect the decline of global strains 48 hours post-surgery, followed by gradual improvement. All global strains were better in the loaded group at 30 days as compared to the control group (p < 0.05 for GLS). In the loaded versus control group, 28% and 32% of patients, respectively, had impaired GLS at baseline. Forty-eight hours after surgery, 92% and 100% of the patients in the loaded and control groups had impaired GLS. Thirty days post-surgery, 36% and 48% of patients in the loaded and control groups, respectively, demonstrated impaired GLS. Left ventricular ejection fraction (LVEF) remained similar between groups throughout the follow-up period.

**Table 2:** Biochemical, strain, and other parameters of patients who underwent OPCABG Data are represented as mean ± SD. p < 0.05 was considered statistically significant. OPCABG: off-pump coronary artery bypass grafting, CK-MB: creatine kinase-MB isoenzyme, GCS: global circumferential strain, GLS: global longitudinal strain, GRS: global radial strain, ICU: intensive care unit, LVEF: left ventricular ejection fraction, NT-proBNP: N-terminal pro-B-type natriuretic peptide, SD: standard deviation

Variables	Loaded group (n = 25)	Control group ( n = 25)	p-value
Hospital stay duration (days)	11.40 ± 1.78	11.32 ± 3.06	0.91
ICU stay duration (hours)	4.32 ± 1.14	4.72 ± 1.72	0.23
Ventilator duration (hours)	14.20 ± 4.42	16.92 ± 7.08	0.11
Inotrope duration (hours)	15.36 ± 6.87	19.28 ± 9.23	0.09
Troponin (ng/mL)			
Baseline	0.50 ± 0.73	0.44 ± 0.61	0.77
At 8 hours	4.28 ± 1.51	5.36 ± 3.22	0.14
At 24 hours	2.66 ± 1.13	4.55 ± 2.12	<0.01
At 48 hours	1.48 ± 0.59	2.00 ± 0.95	0.02
Δ troponin (peak-baseline)	3.88 ± 1.79	5.58 ± 3.12	0.02
CK-MB (IU/L)			
Baseline	4.50 ± 6.76	4.34 ± 6.65	0.93
At 8 hours	27.01 ± 12.39	40.20 ± 18.45	<0.01
At 24 hours	17.76 ± 8.88	27.39 ± 11.11	<0.01
At 48 hours	5.74 ± 3.41	9.86 ± 5.39	<0.01
Δ CK-MB (peak-baseline)	23.01 ± 11.11	38.14 ± 16.51	<0.01
NT-proBNP (pg/mL)			
Baseline	169.28 ± 89.16	167.92 ± 95.86	0.93
At 8 hours	363.60 ± 238.67	624.16 ± 430.54	0.01
At 24 hours	418.48 ± 497.52	905.20 ± 1351.63	0.10
At 48 hours	396.52 ± 865.18	890.40 ± 1795.48	0.22
Δ NT-proBNP (peak-baseline)	244 + 122.10	738.16 + 384.28	<0.01
GLS (%)			
Baseline	-18.92 ± 2.98	-18.40 ± 3.14	0.55
48 hours	-14.08 ± 1.19	-12.00 ± 1.38	<0.01
30 days	-16.80 ± 1.66	-14.28 ± 1.21	<0.01
GCS (%)			
Baseline	-22.24 ± 4.41	-22.16 ± 4.24	0.95
48 hours	-16.48 ± 2.58	-14.76 ± 2.38	0.02
30 days	-18.80 ± 3.24	-17.52 ± 2.76	0.14
GRS (%)			
Baseline	37.84 ± 8.13	37.44 ± 10.51	0.88
48 hours	27.20 ± 6.12	21.92 ± 6.05	<0.01
30 days	31.60 ± 7.84	28.64 ± 7.98	0.19
LVEF (%)			
Baseline	53.64 ± 5.63	53.68 ± 5.33	0.98
48 hours	50.28 ± 4.60	50.16 ± 4.48	0.93
30 days	52.44 ± 4.49	52.56 ± 4.56	0.92

**Figure 2:**
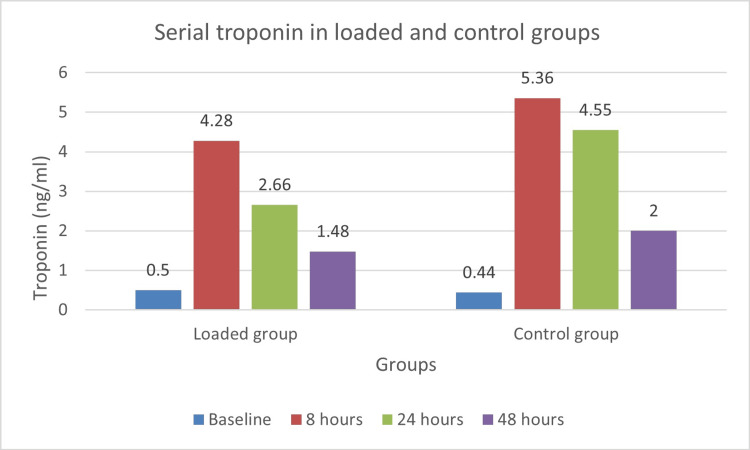
Troponin levels in loaded and control groups at baseline and 8, 24, and 48 hours after OPCABG OPCABG: off-pump coronary artery bypass grafting

The serial measurements of cardiac biomarkers showed distinct patterns between the loaded and control groups. Troponin levels peaked at eight hours post-procedure in both groups; however, the loaded group exhibited lower levels at all subsequent time points after baseline (Figure 2).

**Figure 3:**
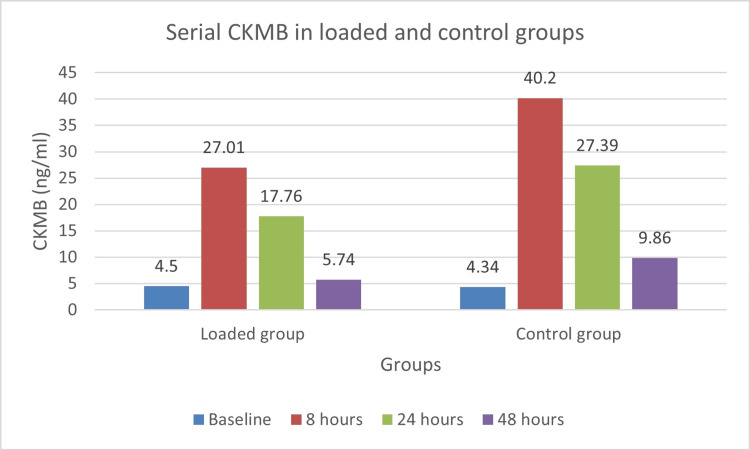
CK-MB levels in loaded and control groups at baseline and 8, 24, and 48 hours after OPCABG OPCABG: off-pump coronary artery bypass grafting, CK-MB: creatine kinase-MB isoenzyme

The difference was particularly notable at 24 hours (2.66 ng/mL versus 4.55 ng/mL) and 48 hours (1.48 ng/mL versus 2.00 ng/mL). CK-MB levels followed a similar trend, with the loaded group consistently showing lower values (Figure 3).

**Figure 4:**
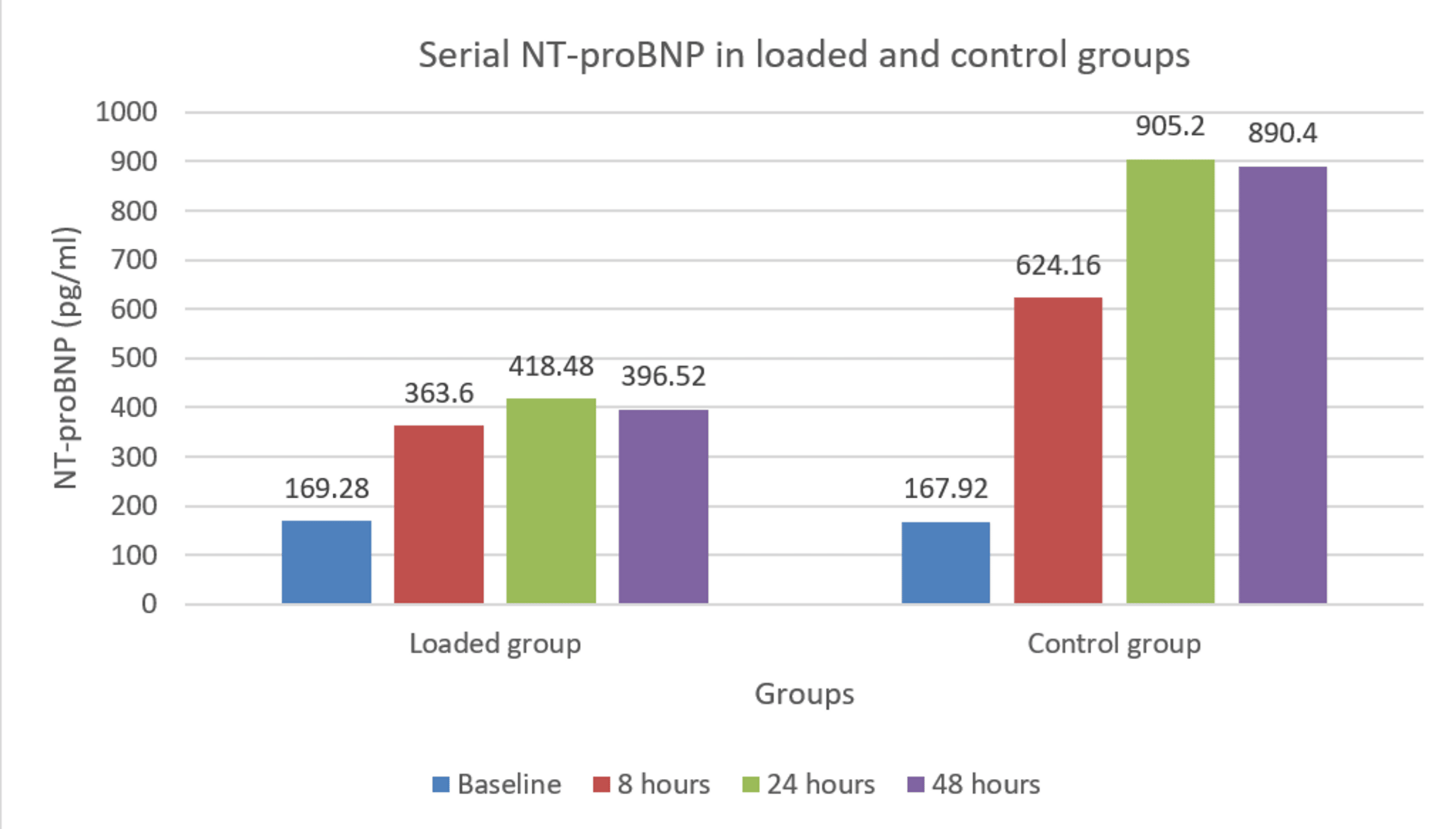
NT-proBNP levels in loaded and control groups at baseline and 8, 24, and 48 hours after OPCABG OPCABG: off-pump coronary artery bypass grafting, NT-proBNP: N-terminal pro-B-type natriuretic peptide

The peak at eight hours was significantly lower in the loaded group (27.01 IU/L) compared to the control group (40.2 IU/L), with this difference persisting at 24 and 48 hours. NT-proBNP levels (Figure 4) had a more pronounced increase in the control group, especially at eight hours (624.16 pg/mL versus 363.6 pg/mL in the loaded group). While both groups showed elevated NT-proBNP levels at 24 and 48 hours compared to baseline, the control group maintained higher levels throughout.

The loaded group had a significantly lower mean delta troponin-I (defined as the change from baseline to peak level) as compared to the control group (3.88 + 1.79 ng/mL versus 5.58 + 3.12 ng/mL, p = 0.02). Similarly, the loaded group had significantly lower mean delta CK-MB (23.01 + 11.11 ng/mL versus 38.14 + 16.51 ng/mL, p < 0.01) and mean delta NT-proBNP (244 + 122.10 pg/mL versus 738.16 + 384.28 pg/mL, p < 0.01).

During the in-hospital course, one patient in the control group died on the seventh postoperative day due to sepsis-induced acute kidney injury. Postoperative atrial fibrillation (AF) occurred in two patients (8%) in the loaded group and six patients (24%) in the control group (p = 0.001). All patients with AF reverted to sinus rhythm with pharmacological management. At one-month follow-up, no additional deaths, myocardial infarctions, repeat revascularizations, or readmissions were observed in either group.

Relationship of strain parameters with postoperative left ventricular ejection fraction

Strain parameters at 48 hours were used to predict 30-day postoperative left ventricular dysfunction. At 48 hours, all three strain parameters were significant predictors of left ventricular ejection fraction (LVEF) ≤ 50% on univariable analysis. On multivariable logistic regression, global circumferential strain (GCS) at 48 hours remained an independent predictor (Table 3).

**Table 3:** Strain parameters predicting 30 days postoperative LVEF ≤ 50% on logistic regression LVEF: left ventricular ejection fraction, CI: confidence interval, GLS: global longitudinal strain, GCS: global circumferential strain, GRS: global radial strain

Variable	Odds ratio	95% CI	p-value
Univariable analysis			
GLS at 48 hours	0.46	0.28-0.77	0.003
GCS at 48 hours	0.27	0.13-0.58	0.001
GRS at 48 hours	0.71	0.59-0.85	<0.001
Multivariable analysis			
GCS at 48 hours	0.28	0.11-0.74	0.010

**Figure 5:**
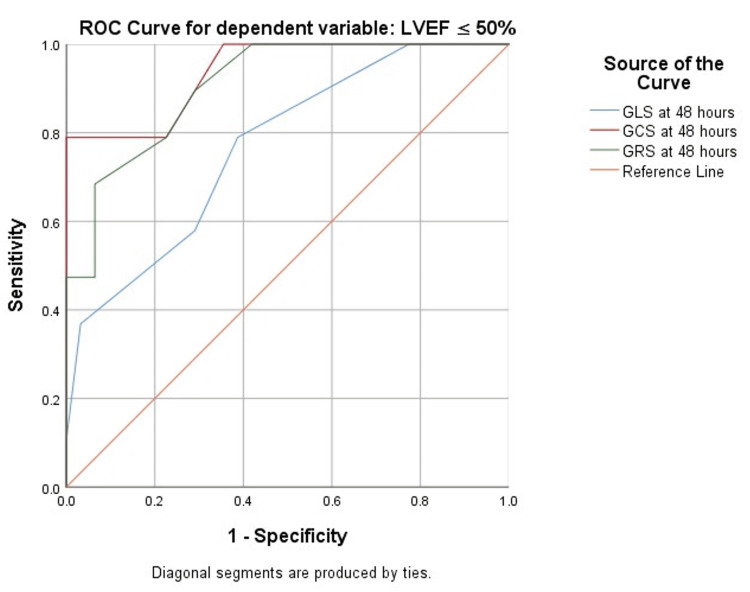
ROC curves for strain parameters at 48 hours to predict 30 days postoperative LVEF ≤ 50% LVEF: left ventricular ejection fraction, ROC: receiver operating characteristic, GLS: global longitudinal strain, GCS: global circumferential strain, GRS: global radial strain

Receiver operating characteristic (ROC) curves were plotted to predict LVEF ≤ 50% using strain parameters at 48 hours (Figure 5). Based on ROC analysis and Youden index, GRS ≤ 26.5 at 48 hours best predicted poor postoperative LVEF at 30 days, with sensitivity of 89.5% and specificity of 71.0%. GLS and GCS were also significant predictors (Table 4).

**Table 4:** ROC analysis of strain parameters to predict 30 days postoperative LVEF ≤ 50% LVEF: left ventricular ejection fraction, ROC: receiver operating characteristic, CI: confidence interval, GLS: global longitudinal strain, GCS: global circumferential strain, GRS: global radial strain

Variable	Cutoff % (test is positive if less than or equal to)	Area under the curve	Sensitivity (%)	Specificity (%)	Youden index	95% CI	p-value
GLS at 48 hours	13.5	0.768	78.9	61.3	0.402	0.64-0.90	0.002
GCS at 48 hours	15.5	0.939	78.9	77.4	0.563	0.88-1.00	<0.001
GRS at 48 hours	26.5	0.907	89.5	71.0	0.605	0.83-0.98	<0.001

**Figure 6:**
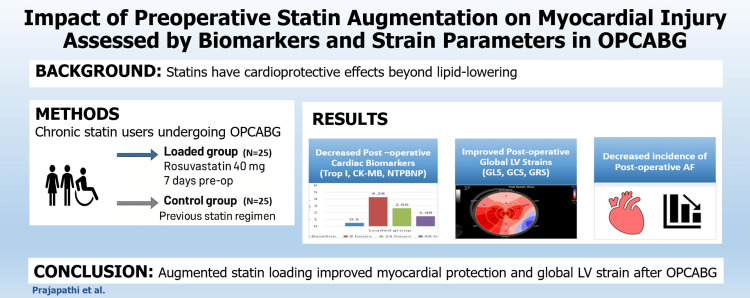
Central illustration OPCABG: off-pump coronary artery bypass grafting, GCS: global circumferential strain, GLS: global longitudinal strain, GRS: global radial strain, LV: left ventricle, NTPBNP: N-terminal pro-B-type natriuretic peptide, CK-MB: creatine kinase-MB isoenzyme, Trop I: troponin I, AF: atrial fibrillation

A schematic illustration summarizing the study flow and key findings is presented in Figure 6.

## Discussion

Our study contributes fresh insights into the association of statin pretreatment with myocardial strain parameters and is among the few to assess changes in all three strain parameters (GLS, GRS, and GCS) in this patient population. All strain parameters declined 48 hours post-surgery, possibly due to perioperative myocardial injury, which was followed by gradual improvement. However, the global LV strains were better in the statin-loaded group at 48 hours (p < 0.05 for GLS, GCS, and GRS) and 30 days post-surgery (p < 0.05 for GLS), suggesting that statin pretreatment may facilitate better myocardial recovery and mechanical function in the postoperative period. On univariable analysis, the strain parameters measured at 48 hours postoperatively were significant predictors of LVEF ≤ 50% at 30 days, concurring with prior studies showing the prognostic value of strain imaging in cardiac surgery. GLS has been consistently reported as a sensitive marker of subclinical myocardial dysfunction, often preceding changes in LVEF. For instance, a study demonstrated that GLS is superior to LVEF in detecting early myocardial dysfunction and is associated with adverse cardiac outcomes across populations [12]. In the context of CABG, it has been reported that impaired preoperative GLS predicts adverse remodeling and postoperative LV dysfunction [13]. Similarly, another study found that early postoperative GLS changes were predictive of long-term LV recovery after revascularization [14].

In our exploratory study, GCS at 48 hours emerged as an independent predictor in multivariable logistic regression. A recent study has also reported baseline GCS ≤ 14% as the best predictor of postoperative LVEF ≤ 50% at 30 days [15]. Although GLS is generally emphasized in clinical practice, circumferential deformation may provide complementary prognostic information, particularly in patients with ischemic cardiomyopathy, where circumferential fibers are preferentially affected [16].

While the predictive role of GRS has been less consistently reported in existing literature due to its high variability and load dependence, a GRS ≤ 26.5 at 48 hours in our exploratory study showed the strongest discriminatory capacity by ROC analysis for 30-day LVEF ≤ 50%, with a sensitivity of 89.5% and specificity of 71.0%. This suggests that radial mechanics may retain clinical utility in the early postoperative period.

Our results support the use of strain imaging for early identification of patients at risk of postoperative LV dysfunction. In our study, the LVEF remained normal throughout the study. A recent review of strain imaging highlights that deformation parameters (including GLS and GCS) have matured in methodology and are increasingly being applied to perioperative and post-surgical settings, emphasizing their ability to detect subtle myocardial injury even when conventional ejection fraction (LVEF) remains preserved, as seen in our study [17]. Another prospective observational study reported that intraoperative and post-induction GLS (in on-pump CABG) were independent predictors of in-hospital mortality, three-month mortality, and postoperative delirium, even in patients with normal LVEF [18]. Strain imaging can be utilized in postoperative monitoring and help in adjusting the treatment course. Future larger-scale trials can validate these findings and explore whether targeted interventions based on early strain assessment could improve clinical outcomes.

Markers of myocardial injury, such as troponins and CK-MB, are known to rise following CABG. Our study aimed to evaluate whether statin pretreatment is associated with the postoperative markers that reflect myocardial injury in such patients. While a previous meta-analysis found insufficient data for aggregate analysis across seven trials, recent studies have suggested that preoperative statin therapy can have beneficial effects on biomarker release kinetics and postoperative outcomes [19-26]. Our observations concur with those of Kaushik et al., who observed reduced levels of troponin, CK-MB, and NT-proBNP in the statin-loaded group as compared to the control group, indicating that statin loading influenced the release kinetics of these biomarkers [11].

Multiple studies have reported a reduction in mortality and arrhythmia incidence post-CABG in patients receiving preoperative statins [27]. A non-randomized retrospective study found statins to be associated with a lower risk of composite clinical outcomes, including death, myocardial infarction, and arrhythmia after CABG [28]. A meta-analysis of 54 studies (including 12 randomized controlled trials) reporting the aggregate data of over 90,000 patients found that preoperative statin therapy was associated with a substantial reduction in early all-cause mortality (p < 0.0001). The same analysis noted significantly reduced odds of new-onset atrial fibrillation [29]. Our study findings coincide with these results. Based on 11 studies included in the review, the mean hospital length of stay for statin-pretreated patients (8.3 ± 2.1 days) was significantly less compared to those without pretreatment (8.8 days ± 2.0 days, p < 0.01). Additionally, the mean ICU stay was significantly shorter in statin-treated patients (2.5 ± 1.4 days) compared to control patients (2.7 ± 1.6 days, p < 0.01) [29]. While our exploratory study’s findings concur with these observations, the results were not statistically significant. Large clinical trials may detect a significant between-group difference favoring the statin-loaded groups and also have implications for healthcare resource utilization and cost-effectiveness. The use of statin loading prior to percutaneous coronary intervention, along with aggressive lipid lowering in post-MI patients, has been recommended [30]. Similarly, more robust evidence can be gathered to recommend statin loading before OPCABG to improve postoperative outcomes. Findings from our exploratory study indicate that the statin-loaded group experienced less myocardial damage and better preservation of cardiac function relative to the control group. Incorporating statin loading into preoperative protocols may improve surgical outcomes and enhance early postoperative recovery in OPCABG patients.

Limitations

This was a single-center observational study conducted over a small sample size and a short follow-up of 30 days. Hence, it was not powered for hard clinical endpoints. Patients being on various statin types, doses, and durations at baseline could have introduced heterogeneity in baseline cardioprotection. This can be seen as both a limitation and a reflection of real-world practice. Additionally, residual perioperative confounding may persist despite standardized institutional protocols. While we acknowledge the limitations of this exploratory study, our findings provide justification for conducting larger, multicentric studies and trials with longer follow-up, which can validate and expand upon these findings. Larger studies researching the mediatory role of inflammatory markers could also provide further insight into the cardioprotective mechanisms.

## Conclusions

Chronic therapy with statins causes attenuation of their pleiotropic mechanisms. Our study suggests that preoperative high-dose rosuvastatin before OPCABG can recapture cardioprotection through pleiotropic effects in patients on chronic statin therapy. The statin-loaded group showed significantly lower postoperative cardiac biomarker levels, improved myocardial strain parameters, faster recovery of LV function, and reduced incidence of atrial fibrillation. Larger multicentric trials can confirm the clinical findings of this study. Statins offer an easy and cost-effective way to reduce ischemia/reperfusion injury during OPCABG. Our study findings may inform the design of future prospective studies evaluating statin-loading strategies for improvement in surgical outcomes and postoperative recovery in OPCABG patients.
